# Nonscalpel Vasectomy as Family Planning Method: A Battle Yet to Be Conquered

**DOI:** 10.1155/2013/752174

**Published:** 2013-04-11

**Authors:** Pankaj Kumar Garg, Bhupendra Kumar Jain, Deepti Choudhary, Ashish Chaurasia, Satya Deo Pandey

**Affiliations:** ^1^Department of Surgery, University College of Medical Sciences and Guru Teg Bahadur Hospital, University of Delhi, Delhi 110095, India; ^2^Department of Obstetrics and Gynaecology, University College of Medical Sciences and Guru Teg Bahadur Hospital, University of Delhi, Delhi 110095, India

## Abstract

Though nonscalpel vasectomy (NSV) technique was introduced in India in 1992 to increase male participation in family planning, it has failed to get adequate momentum and to achieve its goal. We conducted a cross-sectional questionnaire-based survey to get insight into apathy of men towards NSV. The study included 428 respondents. Most of the respondents (97.4%) were aware of NSV as a method for permanent male sterilization. The majority of them (97.2%) knew that NSV is done without any charge and cash incentive is given to the NSV client after the procedure. Though 68.0% respondents agreed that permanent sterilization is a possible option for them, only 34.1% respondents were willing to adopt NSV as a method of family planning. Fear of surgical procedure (40.7%), permanent nature of procedure (22.2%), and religious belief (19.0%) were the common reasons for unwillingness to adopt NSV. We conclude that there is a need to design and develop need-based information, education and communication (IEC) strategy to bridge the existing information gap among the eligible couples regarding NSV to improve its adoption. Involvement of community leaders and satisfied clients and utilization of television and radio would enhance the effectiveness of such interventions.

## 1. Introduction

Nonscalpel vasectomy (NSV) is a modified and sophisticated technique of vasectomy that requires no incision but only a small puncture and no stitches [1]. This is an easier and faster procedure and causes minimal damage to tissues. This is a safe and simple procedure that can be performed in low-resource settings [2]. NSV technique was introduced in India in 1992 to increase male participation in family planning [3]. Despite being a simple and safe method, NSV seems to have failed to achieve its goal. According to the National Family Health Survey-3 (NFHS-3), the current acceptance of NSV in India has declined from 1.9% to 1% in NFHS-2 [4]. This questionnaire-based survey was conducted to get insight into apathy of men towards NSV and their current family planning practices.

## 2. Methods

*Study Design*

 Cross-sectional questionnaire-based survey.

*Setting*

Hospital-based survey in a tertiary teaching hospital of north India.

*Sample Size*

Sample size was calculated using the following formula. Considering 50% awareness of population about NSV (*p* = 0.5), sample size was calculated to be 384 with 95% confidence level and 5% precision of estimate (*d* = 0.05):

(1) $$\begin{array}{c} N=\frac{Z^{2}\left(p\right)\left(1-p\right)}{d^{2}}. \end{array}$$

*Participants*

 Healthy male attendants (aged 18–50 years) of the patients admitted to a tertiary teaching hospital of north India. The participants were married, having at minimum one child, and had not been sterilized previously.

*Questionnaire*

Questionnaire consisted of two parts. First part consisted of eight questions about demographic data (e.g., age of the respondents, age of their wife, number of alive children, age of their youngest child, and information to calculate their Kuppuswamy's socioeconomic status scale [5] (education, occupation, and family income)). No question which could have identified their identity including name, address, caste, or religion was asked. Second part consisted of 13 close-ended questions: eight questions to assess their knowledge about NSV, four to assess their attitude towards male sterilization and NSV, and one to assess if they had been using any contraception. There were two open-ended questions also in the second part of questionnaire: one to assess their present choice of contraception if they had been using, while another question was to know their source of information about NSV.

## 3. Results

The study included 428 respondents who agreed to fill the questionnaires. Mean age of the respondents was 32.43 ± 5.92 years and mean age of their wives was 28.60 ± 5.45. The number of children they had ranged from 1 to 7 (median = 2). The mean age of their youngest child was 4.36 ± 4.30 years. Their mean Kuppuswamy's socioeconomic score was 14.25 ± 4.80, corresponding to lower middle class III.

There were 74.1% respondents who believed that limiting family size will help them stabilize their financial condition. However, only 54.0% respondents were actually practicing the family planning methods. Barrier method and copper T were the most popular methods used by 36.3% and 35.9% respondents using family planning methods, respectively. Tubectomy (female sterilization), oral contraceptive pills, and injectable contraceptives were used by 12.1%, 11.7%, and 3.8% respondents, respectively.

Most of the respondents (97.4%) were aware of vasectomy as a method for permanent male sterilization (Table 1). Television was reported to be their main source of information (47.7% respondents) followed by friends (24.2%). Other sources of information were radio (16.7%), magazines (4.5%), posters (3.5%), newspaper (2.3%), and pamphlets (0.7%). The majority of them (97.2%) knew that NSV is done without any charge and cash incentive is given to the NSV client after the procedure. However, only half of the respondents (50.9%) had knowledge that a monetary compensation is given to the NSV client in case a complication occurs following the procedure, or if the procedure fails. As high as 75.2% respondents were not sure that NSV usually requires one hospital visit only. The fact that NSV does not require prolonged bed rest and does not affect sexual performance was known to 62.9% and 69.6% respondents. Only 66.6% respondents were sure that NSV is done without giving any incision.

A large number of respondents (89.7%) believed that males must also take family planning responsibility. Seven out of ten (68.0%) respondents agreed that permanent sterilization is a possible option for them. However, only 34.1% respondents were willing to adopt NSV as a method of family planning. Reasons cited by the respondents for unwillingness to adopt NSV were fear of surgical procedure (40.7%), permanent nature of procedure (22.2%), religious beliefs (19.0%), decrease in sexual function (10.5%), and requirement of prolonged bed rest following procedure affecting daily earnings (7.5%).

## 4. Discussion

Provisional total of population of India is reported to be 1210.2 million after 2011 census. The population has increased by 181 million during the decade 2001–2010. The percentage decadal growth during 2001–2011 has registered the sharpest decline since independence—a decrease of 3.9 percentage points from 21.54 to 17.64 [6]. However, this decline does not undermine the importance of continued efforts to promote family planning measures. Data from National Family Health Survey- (NFHS-)3 indicated that 56.3% of currently married women were using some method of contraception in 2005-2006 [4]. Our study also corroborates the same as 54.0% respondents in our study confirmed to be practicing some method of contraception. NFHS-3 indicated that only 1% currently married women reported male sterilization as a method of family planning in this survey. This reported use of male sterilization was even worse than indicated by the National Family Health Survey- (NFHS-)2 during 1998-1999. NFHS-2 showed that 1.9% currently married women reported male sterilization as a method of family planning [4]. Almost all the respondents (97.4%) were aware of the NSV as a family planning method. Their source of information was mainly television and friends while printed advertisements (magazines, pamphlets, and posters) hardly contributed anything in spreading knowledge. It seems that circulation of information about family planning by word of mouth is very important.

There has been a general assumption that men, exercising dominations in gender relations, end up shunning of their responsibility of family planning. However, our study provides a new insight as almost nine of ten respondents believed that family planning is also the responsibility of males. It is further important to highlight the fact that only 68.0% of the male respondents approve male sterilization as a possible option of family planning for them. However, only half of them are willing to undergo vasectomy. This highlights the fact that there is large gap in their knowledge about advantages of vasectomy which contribute to their reluctance to undergo vasectomy.

Fear of surgical procedure was cited as the most frequent cause (40.7%) for unwillingness to accept NSV. Many advantages of NSV including no incision, no stitches, and minimal pain were known to only two-thirds of the respondents. It becomes imperative that the procedure should be promoted as simple and painless, and campaign materials should refrain from using the word “operation” in conjunction with NSV. Furthermore, it also merits to be highlighted that a monetary compensation would be given to the client if any complication occurs due to the procedure or in case of failure of the procedure; this fact is known to only half of the respondents (50.9%). Permanent nature of vasectomy was the second common cause (22.2%) for unwillingness to accept NSV. This may be related to the uncertainty about the future. There are a number of reasons for the concern including difficult or impossible remarriage if the current wife dies as person will not be able to father any children after being permanently sterilized, or what he would do if all of his living children died [7–9]. These worries may be overcome by propagating advantages of permanent family planning method, in case family is complete. NSV needs to be propagated as one-time simple and safe solution to the worry felt at the time of intercourse. Awareness of problems associated with other family planning methods (side effects of intrauterine device, associated uneasiness with condoms, and problem of daily intake of oral contraceptive pills) may prove a trigger for promoting a person to take a final decision about NSV [10]. Religious reasons were cited by the respondents as a third common barrier to NSV uptake. It may definitely be inferred that involvement of community leaders may enhance the acceptability of NSV. Requirement of prolonged bed rest after vasectomy was another important reason cited by the respondents for their reluctance to adopt NSV. This is highlighted by the fact that only 37.1% respondents were not of clear opinion that NSV does not require prolonged bed rest. This aspect is likely to be important for the people who work on the basis of daily wages. Promotional activities should specifically highlight this important issue that the clients may join their work the next day following the NSV. Worry about the impact on sexual life following NSV was another barrier to NSV uptake. A large number of respondents (31.4%) were not sure that sexual performance would not be affected following NSV. This aspect was also highlighted in another survey where it was noted that “men would not tell other people if they had been sterilized, fearing being shamed and taunted by community members, who might refer to them using such words as *namard *(meaning infertile)” [10]. In a survey conducted to study the factors affecting vasectomy acceptability in the Kigoma region of Tanzania, it was pointed out rumors that vasectomy results in decreased sexual desire or performance or that the procedure is equivalent to castration were prevalent and were mentioned by respondents [11].

In conclusion, the NSV promotional activities should focus on bridging the prevailing information gap regarding NSV among the potential clients. A client satisfied with NSV may prove instrumental in convincing other persons to opt for NSV. This has been very aptly narrated by Dr. R.C.M. Kaza, NSV Master Trainer to the Government of India as follows: “NSV is as much an IEC operation as a surgical operation” [12]. Airing positive stories and examples of successful NSV cases through the powerful media of television is likely to improve the acceptability of NSV among the masses.

There is a need to design and develop a need-based IEC strategy to bridge the existing information gap among the eligible couples regarding NSV to improve its adoption. Involvement of community leaders and satisfied clients in the promotional activities and utilization of television and radio would enhance the effectiveness of such interventions.

## Figures and Tables

**Table 1:** KAP study on nonscalpel vasectomy and family planning.

	Yes	No	Cannot say
Knowledge			
Are you aware that vasectomy is an option for permanent sterilization?	417 (97.4%)	9 (2.1%)	2 (0.4%)
Are you aware that vasectomy is done without any charge?	416 (97.2%)	4 (0.9%)	8 (1.8%)
Are you aware that cash incentive is given after vasectomy?	392 (91.6%)	25 (5.8%)	11 (2.6%)
Are you aware that there is provision for insurance if pregnancy or any other complication occurs after vasectomy?	218 (50.9%)	98 (22.9%)	112 (26.2%)
Are you aware that vasectomy is done in one OPD visit only?	106 (24.8%)	221 (51.6%)	101 (23.6%)
Are you aware that vasectomy does not require prolonged bed rest?	269 (62.9%)	111 (25.9%)	48 (11.2%)
Are you aware that vasectomy does not affect sexual performance?	298 (69.6%)	83 (19.4%)	47 (11.0%)
Are you aware that vasectomy is done without giving any incision?	285 (66.6%)	123 (28.7%)	20 (4.7)

Attitude			
Do you think limiting family size is an option to stabilize financial condition?	317 (74.1%)	78 (18.2%)	33 (7.7%)
Do you think family planning is also responsibility of males?	384 (89.7%)	30 (7.0%)	14 (3.3%)
Do you find permanent sterilization a possible option for yourself?	291 (68.0%)	64 (15.0%)	73 (17.0%)
Are you willing to adopt vasectomy as a method of family planning?	146 (34.1%)	189 (44.2%)	93 (21.7%)

Practice			
Are you practicing any family planning measure?	231 (54.0%)	197 (46.0%)	
